# *N*-(2,6-Dimethyl-3-oxo-1-thia-4-aza­spiro­[4.5]dec-4-yl)-2-hydr­oxy-2,2-di­phenyl­acetamide

**DOI:** 10.1107/S1600536808028651

**Published:** 2008-09-13

**Authors:** Şerife Pınar Yalçın, Mehmet Akkurt, Ertan Şahin, Özlen Güzel, Aydın Salman, Eser İhan

**Affiliations:** a Department of Physics, Faculty of Arts and Sciences, Erciyes University, 38039 Kayseri, Turkey; b Department of Chemistry, Faculty of Arts and Sciences, Atatürk University, 22240 Erzurum, Turkey; c Department of Pharmaceutical Chemistry, Faculty of Pharmacy, Istanbul University, 34116 Istanbul, Turkey

## Abstract

In the title compound, C_24_H_28_N_2_O_3_S, the pendant methyl C atom bonded to the cyclo­hexane ring is disordered over two sites in a 0.580 (11):0.420 (11) ratio. The cyclo­hexane ring adopts a distorted chair conformation while the thia­zolidine ring has an envelope conformation. The two phenyl rings make a dihedral angle of 71.8 (2)° with each other. The conformation is stabilized by an intra­molecular N—H⋯O hydrogen bond. In the crystal structure, an inter­molecular hydrogen bond O—H⋯O occurs.

## Related literature

For background, see: Güzel *et al.* (2006 ▶). For a related structure, see: Akkurt *et al.* (2007 ▶). For ring puckering parameters, see: Cremer & Pople (1975 ▶).
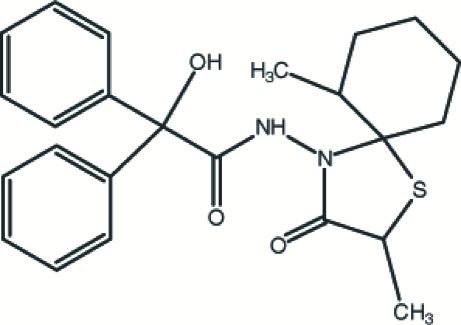


## Experimental

### 

#### Crystal data

C_24_H_28_N_2_O_3_S
*M* *_r_* = 423.55
Monoclinic, 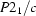

*a* = 9.4942 (4) Å
*b* = 20.6765 (5) Å
*c* = 12.0052 (4) Å
β = 105.063 (2)°
*V* = 2275.73 (14) Å^3^
*Z* = 4
Mo *K*α radiation
μ = 0.17 mm^−1^
*T* = 293.1 K
0.20 × 0.20 × 0.20 mm

#### Data collection

Rigaku R-AXIS conversion diffractometer
Absorption correction: empirical (using intensity measurements)*XABS2* (Parkin *et al*., 1995 ▶) *T*_min_ = 0.967, *T*_max_ = 0.967
6931 measured reflections
6931 independent reflections
3002 reflections with *I* > 2σ(*I*)

#### Refinement

*R*[*F* ^2^ > 2σ(*F* ^2^)] = 0.094
*wR*(*F* ^2^) = 0.235
*S* = 1.05
6931 reflections
301 parameters
H atoms treated by a mixture of independent and constrained refinement
Δρ_max_ = 0.28 e Å^−3^
Δρ_min_ = −0.35 e Å^−3^

### 

Data collection: *CrystalClear* (Rigaku/MSC, 2005 ▶); cell refinement: *CrystalClear* data reduction: *CrystalClear*; program(s) used to solve structure: *SIR97* (Altomare *et al.*, 1999 ▶); program(s) used to refine structure: *SHELXL97* (Sheldrick, 2008 ▶); molecular graphics: *ORTEP-3 for Windows* (Farrugia, 1997 ▶); software used to prepare material for publication: *PLATON* (Spek, 2003 ▶).

## Supplementary Material

Crystal structure: contains datablocks global, I. DOI: 10.1107/S1600536808028651/hb2789sup1.cif

Structure factors: contains datablocks I. DOI: 10.1107/S1600536808028651/hb2789Isup2.hkl

Additional supplementary materials:  crystallographic information; 3D view; checkCIF report

## Figures and Tables

**Table 1:** Hydrogen-bond geometry (Å, °)

*D*—H⋯*A*	*D*—H	H⋯*A*	*D*⋯*A*	*D*—H⋯*A*
O3—H*O*3⋯O1^i^	0.92 (5)	1.83 (5)	2.744 (4)	175 (5)
N2—H*N*2⋯O3	0.86 (3)	2.11 (3)	2.535 (4)	110 (3)

Symmetry code: (i) 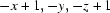.

**Fig. 1.**
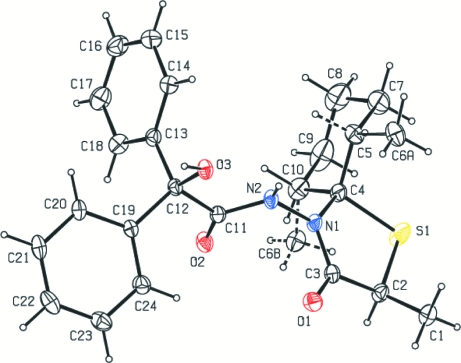
View of the molecular structure of (I) showing 10% displacement ellipsoids for the non-hydrogen atoms.

**Fig. 2.**
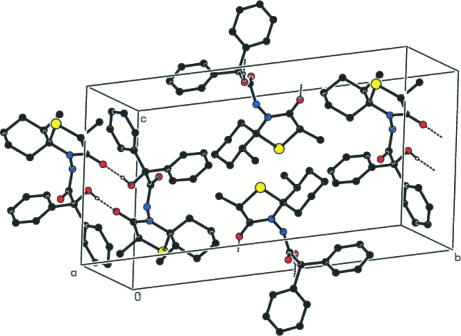
Packing for (I) with hydrogen bonds shown as dashed lines. The H atoms not involved in hydrogen contacts have been omitted.

## supplementary crystallographic information

### Comment

In our previous report (Güzel *et al.*, 2006), we described the synthesis and evaluation of sixteen new2-hydroxy-*N*-(3-oxo-1-thia-4-azaspiro[4.4]non-4-yl)/(3-oxo-1-thia-4-azaspiro[4.5]dec-4-yl)-2,2-diphenylacetamidederivatives, as potential antimicro-bacterial agents. We now report the crystal structure of the related title compound, (I), (Fig. 1).

The geometric parameters in (I) are comparable with those in 2-hydroxy-*N*-(3-oxo-1-thia-4-azaspiro[4.5]dec-4-yl)-2,2-diphenylacetamide (Akkurt *et al.*, 2007). The dihedral angle between the two phenyl rings (C13—C18) and (C19—C24) in (I) is 71.8 (2)°. The five-membered ring (S1/C2/C3/N1/C4) has an envelope conformation with S1 at the flap position [puckering parameters: Q_2_ = 0.167 (3)Å and φ_2_ = 352.2 (12)° (Cremer & Pople, 1975)]. The cyclohexane ring has a distorted chair conformation, with the puckering parameters: Q = 0.585 (7) Å, θ = 2.7 (7) and φ = 132 (14)°.

The molecular conformation and crystal packing are stabilized by N—H···O and O—H···O hydrogen bonding interactions (Table 1).

### Experimental

A mixture of 2-hydroxy-2,2-diphenylacetohydrazide (0.005 mol), 2-methylcyclohexanone (0.005 mol) and mercaptoacetic acid or α-mercaptopropionic acid (0.02 mol) was refluxed in 20 ml dry benzene for 5–6 h using a Dean-Stark water separator. Excess benzene was evaporated *in vacuo*. The resulting residue was triturated with saturated NaHCO_3_ solution until CO_2_ evolution ceased and was allowed to stand overnight or in some cases refrigerated until solidification. The solid thus obtained was washed with water, dried, and recrystallized from ethanol. [Yield 48%, mp 455–458 K]. IR(KBr) (ν, cm^-1^): 3335 (O—H/N—H), 1679,1731 (C=O); ^1^H-NMR (DMSO-d_6_, 500 MHz) δ (p.p.m.): 0.93 (t, 3H, J = 6.83 Hz, 6-CH_3_), 0.95–1.10 (m, 1H, spirodecane),1.11–1.14 (m, 1H, spirodecane), 1.19–1.27 (m, 1H, spirodecane),1.41 (t, 3H, J = 7.32 Hz, 2-CH_3_), 1.45–1.67 (m, 5H, spirodecane), 1.83–1.87 (m, 1H, spirodecane), 3.77,3.85 (2q, 1H, J = 6.83 Hz, C_2_—H), 6.78 (s, 1H, COH), 7.26–7.31 (m, 2H, Ar—H), 7.33–7.35 (m, 4H, Ar—H), 7.45–7.49 (m, 4H,Ar—H), 9.93,10.05 (2 s, 1H, CONH). Analysis calculated for C_24_H_28_N_2_O_3_S: C 67.90, H 6.65, N 6.60%. Found: C 68.27, H 6.89, N 6.24%.

### Refinement

The methyl C atom bonded to the cyclohexane ring is disordered over two sites in a 0.580 (11):0.420 (11) ratio.  The attached H atoms were located in difference maps and refined with the same fractional occupancies as their carrier carbon atoms and a fixed *U*_iso_ value of 0.05 Å^2^. The hydroxyl and amine H atoms were found from difference maps and refined freely. The other H atoms were located geometrically and constrained to ride on their parent atoms with C–H = 0.93-0.97 Å,and with *U*_iso_(H) = 1.2U_eq_(C)or *U*_eq_(methyl C).

### Crystal data

**Table:**

C_24_H_28_N_2_O_3_S	*F*(000) = 900
*M**_r_* = 423.55	*D*_x_ = 1.236 Mg m^−^^3^
Monoclinic, *P*2_1_/*c*	Mo *K*α radiation, λ = 0.7107 Å
Hall symbol: -P 2ybc	Cell parameters from 4481 reflections
*a* = 9.4942 (4) Å	θ = 2.4–30.6°
*b* = 20.6765 (5) Å	µ = 0.17 mm^−^^1^
*c* = 12.0052 (4) Å	*T* = 293 K
β = 105.063 (2)°	Block, pale yellow
*V* = 2275.73 (14) Å^3^	0.20 × 0.20 × 0.20 mm
*Z* = 4	

### Data collection

**Table:**

Rigaku R-AXIS conversion diffractometer	6931 independent reflections
Radiation source: Sealed Tube	3002 reflections with *I* > 2σ(*I*)
Graphite Monochromator	*R*_int_ = 0.000
Detector resolution: 10.0000 pixels mm^-1^	θ_max_ = 30.5°, θ_min_ = 2.6°
dtprofit.ref scans	*h* = −13→13
Absorption correction: empirical (using intensity measurements) (using intensity measurements) XABS2 (Parkin et al., 1995)	*k* = 0→29
*T*_min_ = 0.967, *T*_max_ = 0.967	*l* = 0→17
6931 measured reflections	

### Refinement

**Table:**

Refinement on *F*^2^	Primary atom site location: structure-invariant direct methods
Least-squares matrix: full	Secondary atom site location: difference Fourier map
*R*[*F*^2^ > 2σ(*F*^2^)] = 0.094	Hydrogen site location: difmap and geom
*wR*(*F*^2^) = 0.235	H atoms treated by a mixture of independent and constrained refinement
*S* = 1.05	*w* = 1/[σ^2^(*F*_o_^2^) + (0.0588*P*)^2^ + 1.0272*P*] where *P* = (*F*_o_^2^ + 2*F*_c_^2^)/3
6931 reflections	(Δ/σ)_max_ < 0.001
301 parameters	Δρ_max_ = 0.28 e Å^−^^3^
0 restraints	Δρ_min_ = −0.35 e Å^−^^3^

### Special details

**Table:**

Geometry. Bond distances, angles *etc*. have been calculated using the rounded fractional coordinates. All su's are estimated from the variances of the (full) variance-covariance matrix. The cell e.s.d.'s are taken into account in the estimation of distances, angles and torsion angles
Refinement. Refinement on *F*^2^ for ALL reflections except those flagged by the user for potential systematic errors. Weighted *R*-factors *wR* and all goodnesses of fit *S* are based on *F*^2^, conventional *R*-factors *R* are based on *F*, with *F* set to zero for negative *F*^2^. The observed criterion of *F*^2^ > σ(*F*^2^) is used only for calculating -*R*-factor-obs *etc*. and is not relevant to the choice of reflections for refinement. *R*-factors based on *F*^2^ are statistically about twice as large as those based on *F*, and *R*-factors based on ALL data will be even larger.

### Fractional atomic coordinates and isotropic or equivalent isotropic displacement parameters (Å^2^)

**Table:**

	*x*	*y*	*z*	*U*_iso_*/*U*_eq_	Occ. (<1)
S1	−0.11370 (14)	0.07379 (6)	0.21004 (13)	0.1429 (6)	
O1	0.1607 (3)	−0.03247 (12)	0.4220 (2)	0.0880 (10)	
O2	0.2399 (2)	0.11034 (14)	0.5737 (2)	0.0862 (9)	
O3	0.5610 (3)	0.07766 (12)	0.48919 (19)	0.0738 (9)	
N1	0.1428 (3)	0.06961 (14)	0.3499 (2)	0.0709 (10)	
N2	0.2874 (3)	0.08385 (15)	0.4045 (2)	0.0713 (10)	
C1	−0.0926 (4)	−0.0586 (2)	0.2264 (4)	0.0989 (17)	
C2	−0.0673 (3)	0.00255 (17)	0.2971 (3)	0.0772 (11)	
C3	0.0903 (3)	0.01066 (18)	0.3637 (3)	0.0698 (11)	
C4	0.0490 (4)	0.11999 (18)	0.2824 (3)	0.0791 (12)	
C5	0.1203 (7)	0.1502 (3)	0.1956 (4)	0.109 (2)	
C6A	0.1402 (11)	0.1054 (5)	0.1149 (8)	0.109 (4)	0.580 (11)
C7	0.0264 (9)	0.2027 (3)	0.1258 (6)	0.188 (4)	
C8	0.0013 (10)	0.2558 (4)	0.2017 (8)	0.212 (5)	
C9	−0.0740 (7)	0.2280 (3)	0.2883 (7)	0.170 (3)	
C10	0.0158 (6)	0.1737 (3)	0.3578 (6)	0.106 (2)	
C11	0.3265 (3)	0.09945 (16)	0.5184 (3)	0.0671 (11)	
C12	0.4926 (3)	0.10928 (16)	0.5668 (3)	0.0630 (10)	
C13	0.5154 (3)	0.18202 (17)	0.5640 (3)	0.0689 (11)	
C14	0.5762 (5)	0.2092 (2)	0.4832 (3)	0.0970 (17)	
C15	0.5908 (6)	0.2758 (3)	0.4764 (4)	0.123 (3)	
C16	0.5463 (6)	0.3152 (2)	0.5502 (5)	0.122 (2)	
C17	0.4866 (5)	0.2891 (2)	0.6331 (5)	0.115 (2)	
C18	0.4700 (4)	0.2227 (2)	0.6395 (4)	0.0906 (17)	
C19	0.5443 (3)	0.07960 (16)	0.6865 (3)	0.0636 (11)	
C20	0.6706 (4)	0.10250 (19)	0.7626 (3)	0.0812 (14)	
C21	0.7222 (5)	0.0740 (3)	0.8703 (3)	0.1008 (18)	
C22	0.6495 (6)	0.0232 (3)	0.9013 (4)	0.111 (2)	
C23	0.5267 (5)	−0.0006 (2)	0.8266 (4)	0.1013 (17)	
C24	0.4747 (4)	0.02714 (19)	0.7194 (3)	0.0827 (14)	
C6B	−0.0810 (13)	0.1490 (6)	0.4150 (10)	0.092 (4)	0.420 (11)
HO3	0.656 (5)	0.065 (2)	0.521 (4)	0.117 (15)*	
H1A	−0.19400	−0.06190	0.18650	0.1480*	
H1C	−0.03520	−0.05760	0.17130	0.1480*	
H2	−0.12760	0.00110	0.35200	0.0920*	
H1B	−0.06470	−0.09530	0.27630	0.1480*	
H6A1	0.19960	0.07040	0.15370	0.1640*	0.580 (11)
H6A2	0.18750	0.12590	0.06270	0.1640*	0.580 (11)
H6A3	0.04710	0.08890	0.07260	0.1640*	0.580 (11)
H7A	0.07390	0.21990	0.06970	0.2260*	
H7B	−0.06660	0.18450	0.08420	0.2260*	
H8A	−0.05930	0.28900	0.15580	0.2550*	
HN2	0.353 (3)	0.0683 (14)	0.374 (3)	0.060 (10)*	
H9A	−0.16920	0.21150	0.24780	0.2030*	
H9B	−0.08800	0.26190	0.34010	0.2030*	
H10A	0.115 (6)	0.193 (2)	0.400 (4)	0.0500*	0.580 (11)
H10B	−0.010 (8)	0.156 (4)	0.410 (6)	0.0500*	0.580 (11)
H14	0.60820	0.18270	0.43220	0.1160*	
H15	0.63160	0.29340	0.42060	0.1470*	
H16	0.55580	0.35980	0.54500	0.1460*	
H17	0.45730	0.31610	0.68490	0.1380*	
H18	0.42810	0.20530	0.69490	0.1090*	
H20	0.72090	0.13700	0.74150	0.0970*	
H21	0.80650	0.08960	0.92130	0.1210*	
H22	0.68370	0.00460	0.97380	0.1320*	
H23	0.47800	−0.03560	0.84810	0.1210*	
H24	0.39170	0.01030	0.66850	0.0990*	
H8B	0.09360	0.27520	0.24180	0.2550*	
H5A	0.229 (7)	0.170 (3)	0.247 (5)	0.0500*	0.420 (11)
H5B	0.175 (9)	0.122 (5)	0.171 (9)	0.0500*	0.420 (11)
H6B1	−0.10350	0.18160	0.46470	0.1380*	0.420 (11)
H6B2	−0.03800	0.11230	0.46020	0.1380*	0.420 (11)
H6B3	−0.16890	0.13590	0.35980	0.1380*	0.420 (11)

### Atomic displacement parameters (Å^2^)

**Table:**

	*U*^11^	*U*^22^	*U*^33^	*U*^12^	*U*^13^	*U*^23^
S1	0.1011 (9)	0.1045 (9)	0.1652 (13)	−0.0240 (7)	−0.0691 (8)	0.0456 (8)
O1	0.0723 (15)	0.0910 (18)	0.0904 (18)	0.0132 (13)	0.0026 (13)	0.0235 (14)
O2	0.0574 (13)	0.126 (2)	0.0696 (15)	0.0027 (14)	0.0067 (12)	0.0014 (14)
O3	0.0580 (13)	0.0954 (18)	0.0620 (14)	0.0095 (12)	0.0050 (11)	−0.0062 (12)
N1	0.0575 (15)	0.0745 (18)	0.0670 (17)	−0.0033 (13)	−0.0084 (12)	0.0063 (14)
N2	0.0517 (15)	0.095 (2)	0.0589 (17)	−0.0003 (15)	−0.0007 (13)	−0.0060 (15)
C1	0.089 (3)	0.100 (3)	0.098 (3)	−0.013 (2)	0.007 (2)	−0.015 (2)
C2	0.0580 (19)	0.081 (2)	0.084 (2)	−0.0039 (17)	0.0028 (16)	−0.0012 (19)
C3	0.0591 (19)	0.080 (2)	0.063 (2)	0.0074 (17)	0.0028 (15)	0.0009 (17)
C4	0.065 (2)	0.078 (2)	0.078 (2)	−0.0025 (18)	−0.0107 (17)	0.0153 (19)
C5	0.132 (4)	0.104 (4)	0.079 (3)	−0.007 (3)	0.005 (3)	0.019 (3)
C6A	0.106 (6)	0.139 (8)	0.082 (6)	0.030 (5)	0.023 (5)	−0.006 (5)
C7	0.239 (8)	0.132 (5)	0.135 (5)	−0.041 (5)	−0.056 (5)	0.063 (4)
C8	0.227 (9)	0.107 (5)	0.225 (9)	−0.023 (5)	−0.081 (7)	0.060 (6)
C9	0.136 (5)	0.098 (4)	0.218 (7)	0.037 (4)	−0.056 (5)	−0.016 (4)
C10	0.085 (3)	0.088 (3)	0.129 (5)	0.016 (3)	0.002 (3)	0.009 (3)
C11	0.0555 (18)	0.075 (2)	0.064 (2)	0.0002 (15)	0.0035 (15)	0.0074 (16)
C12	0.0567 (17)	0.075 (2)	0.0529 (17)	0.0009 (15)	0.0063 (14)	0.0008 (15)
C13	0.0609 (19)	0.074 (2)	0.065 (2)	0.0010 (16)	0.0040 (15)	−0.0002 (17)
C14	0.116 (3)	0.093 (3)	0.080 (3)	−0.022 (2)	0.022 (2)	0.001 (2)
C15	0.162 (5)	0.101 (4)	0.101 (4)	−0.033 (3)	0.027 (3)	0.009 (3)
C16	0.139 (4)	0.079 (3)	0.135 (5)	−0.014 (3)	0.012 (4)	0.004 (3)
C17	0.115 (4)	0.089 (3)	0.136 (4)	0.004 (3)	0.025 (3)	−0.023 (3)
C18	0.084 (3)	0.084 (3)	0.104 (3)	0.003 (2)	0.025 (2)	−0.007 (2)
C19	0.0625 (18)	0.074 (2)	0.0508 (17)	0.0061 (16)	0.0085 (14)	−0.0003 (15)
C20	0.075 (2)	0.097 (3)	0.060 (2)	−0.0005 (19)	−0.0035 (17)	−0.0072 (18)
C21	0.091 (3)	0.132 (4)	0.061 (2)	0.016 (3)	−0.013 (2)	−0.008 (2)
C22	0.122 (4)	0.136 (4)	0.065 (3)	0.036 (3)	0.009 (3)	0.022 (3)
C23	0.107 (3)	0.107 (3)	0.087 (3)	0.014 (3)	0.020 (3)	0.029 (2)
C24	0.077 (2)	0.089 (3)	0.076 (2)	0.007 (2)	0.0089 (18)	0.010 (2)
C6B	0.070 (7)	0.101 (8)	0.107 (8)	0.002 (6)	0.025 (6)	−0.021 (6)

### Geometric parameters (Å, °)

**Table:**

S1—C2	1.794 (4)	C21—C22	1.361 (8)
S1—C4	1.832 (4)	C22—C23	1.365 (7)
O1—C3	1.220 (4)	C23—C24	1.378 (6)
O2—C11	1.205 (4)	C1—H1A	0.9600
O3—C12	1.425 (4)	C1—H1B	0.9600
O3—HO3	0.92 (5)	C1—H1C	0.9600
N1—C3	1.343 (5)	C2—H2	0.9800
N1—C4	1.470 (5)	C5—H5A	1.13 (7)
N1—N2	1.391 (4)	C5—H5B	0.88 (9)
N2—C11	1.359 (4)	C6A—H6A1	0.9600
N2—HN2	0.86 (3)	C6A—H6A2	0.9600
C1—C2	1.507 (6)	C6A—H6A3	0.9600
C2—C3	1.511 (4)	C6B—H6B1	0.9600
C4—C5	1.517 (7)	C6B—H6B2	0.9600
C4—C10	1.517 (7)	C6B—H6B3	0.9600
C5—C7	1.512 (9)	C7—H7A	0.9700
C5—C6A	1.388 (11)	C7—H7B	0.9700
C6B—C10	1.380 (14)	C8—H8A	0.9700
C7—C8	1.485 (11)	C8—H8B	0.9700
C8—C9	1.519 (12)	C9—H9B	0.9700
C9—C10	1.521 (9)	C9—H9A	0.9700
C11—C12	1.546 (4)	C10—H10B	0.82 (7)
C12—C19	1.522 (5)	C10—H10A	1.03 (5)
C12—C13	1.521 (5)	C14—H14	0.9300
C13—C18	1.385 (5)	C15—H15	0.9300
C13—C14	1.372 (5)	C16—H16	0.9300
C14—C15	1.389 (7)	C17—H17	0.9300
C15—C16	1.350 (8)	C18—H18	0.9300
C16—C17	1.377 (8)	C20—H20	0.9300
C17—C18	1.386 (6)	C21—H21	0.9300
C19—C24	1.380 (5)	C22—H22	0.9300
C19—C20	1.388 (5)	C23—H23	0.9300
C20—C21	1.389 (5)	C24—H24	0.9300
			
C2—S1—C4	95.90 (17)	S1—C2—H2	109.00
C12—O3—HO3	115 (3)	C1—C2—H2	109.00
N2—N1—C3	119.1 (3)	C3—C2—H2	109.00
C3—N1—C4	121.3 (3)	C4—C5—H5A	106 (3)
N2—N1—C4	119.5 (3)	C4—C5—H5B	111 (7)
N1—N2—C11	120.2 (3)	C7—C5—H5A	111 (3)
N1—N2—HN2	117 (2)	C7—C5—H5B	127 (7)
C11—N2—HN2	119 (2)	H5A—C5—H5B	84 (7)
C1—C2—C3	112.5 (3)	C5—C6A—H6A1	109.00
S1—C2—C1	112.5 (3)	C5—C6A—H6A2	109.00
S1—C2—C3	106.1 (2)	C5—C6A—H6A3	109.00
O1—C3—C2	122.8 (3)	H6A1—C6A—H6A2	110.00
O1—C3—N1	124.6 (3)	H6A1—C6A—H6A3	109.00
N1—C3—C2	112.6 (3)	H6A2—C6A—H6A3	110.00
S1—C4—N1	102.0 (2)	H6B2—C6B—H6B3	109.00
S1—C4—C5	111.2 (3)	C10—C6B—H6B3	109.00
C5—C4—C10	107.9 (4)	C10—C6B—H6B1	109.00
S1—C4—C10	112.6 (3)	C10—C6B—H6B2	109.00
N1—C4—C5	110.9 (4)	H6B1—C6B—H6B2	110.00
N1—C4—C10	112.3 (3)	H6B1—C6B—H6B3	109.00
C4—C5—C6A	111.8 (6)	C5—C7—H7B	109.00
C4—C5—C7	111.9 (5)	C5—C7—H7A	110.00
C6A—C5—C7	105.1 (6)	H7A—C7—H7B	108.00
C5—C7—C8	110.9 (6)	C8—C7—H7A	109.00
C7—C8—C9	108.6 (6)	C8—C7—H7B	109.00
C8—C9—C10	111.0 (6)	C9—C8—H8B	110.00
C6B—C10—C9	101.3 (7)	C7—C8—H8A	110.00
C4—C10—C6B	107.1 (7)	C7—C8—H8B	110.00
C4—C10—C9	112.8 (5)	C9—C8—H8A	110.00
O2—C11—N2	123.5 (3)	H8A—C8—H8B	108.00
O2—C11—C12	123.0 (3)	C8—C9—H9A	109.00
N2—C11—C12	113.2 (3)	C8—C9—H9B	109.00
C11—C12—C13	105.0 (3)	H9A—C9—H9B	108.00
C11—C12—C19	110.4 (3)	C10—C9—H9B	109.00
C13—C12—C19	114.0 (3)	C10—C9—H9A	109.00
O3—C12—C19	110.0 (3)	C9—C10—H10A	107 (2)
O3—C12—C11	106.7 (3)	C4—C10—H10B	106 (6)
O3—C12—C13	110.3 (3)	C4—C10—H10A	106 (3)
C12—C13—C14	120.6 (3)	C9—C10—H10B	121 (6)
C14—C13—C18	118.3 (3)	H10A—C10—H10B	102 (6)
C12—C13—C18	121.1 (3)	C13—C14—H14	120.00
C13—C14—C15	121.0 (4)	C15—C14—H14	119.00
C14—C15—C16	120.5 (5)	C16—C15—H15	120.00
C15—C16—C17	119.7 (4)	C14—C15—H15	120.00
C16—C17—C18	120.2 (4)	C15—C16—H16	120.00
C13—C18—C17	120.4 (4)	C17—C16—H16	120.00
C12—C19—C20	119.7 (3)	C16—C17—H17	120.00
C12—C19—C24	121.8 (3)	C18—C17—H17	120.00
C20—C19—C24	118.4 (3)	C17—C18—H18	120.00
C19—C20—C21	120.2 (4)	C13—C18—H18	120.00
C20—C21—C22	120.1 (4)	C19—C20—H20	120.00
C21—C22—C23	120.4 (4)	C21—C20—H20	120.00
C22—C23—C24	120.1 (4)	C20—C21—H21	120.00
C19—C24—C23	120.8 (4)	C22—C21—H21	120.00
C2—C1—H1A	109.00	C23—C22—H22	120.00
C2—C1—H1B	110.00	C21—C22—H22	120.00
C2—C1—H1C	110.00	C22—C23—H23	120.00
H1A—C1—H1B	109.00	C24—C23—H23	120.00
H1A—C1—H1C	109.00	C23—C24—H24	120.00
H1B—C1—H1C	109.00	C19—C24—H24	120.00
			
C4—S1—C2—C1	−135.6 (3)	C8—C9—C10—C4	−56.3 (7)
C4—S1—C2—C3	−12.3 (3)	O2—C11—C12—O3	166.6 (3)
C2—S1—C4—N1	13.4 (2)	O2—C11—C12—C13	−76.3 (4)
C2—S1—C4—C5	131.6 (3)	O2—C11—C12—C19	47.0 (4)
C2—S1—C4—C10	−107.2 (4)	N2—C11—C12—O3	−19.8 (4)
C3—N1—N2—C11	−79.3 (4)	N2—C11—C12—C13	97.4 (3)
C4—N1—N2—C11	98.2 (4)	N2—C11—C12—C19	−139.3 (3)
N2—N1—C3—O1	0.3 (5)	O3—C12—C13—C14	8.4 (4)
N2—N1—C3—C2	−178.9 (3)	O3—C12—C13—C18	−174.3 (3)
C4—N1—C3—O1	−177.2 (3)	C11—C12—C13—C14	−106.2 (4)
C4—N1—C3—C2	3.7 (4)	C11—C12—C13—C18	71.1 (4)
N2—N1—C4—S1	170.4 (2)	C19—C12—C13—C14	132.8 (3)
N2—N1—C4—C5	52.0 (4)	C19—C12—C13—C18	−49.9 (4)
N2—N1—C4—C10	−68.8 (4)	O3—C12—C19—C20	86.8 (4)
C3—N1—C4—S1	−12.2 (4)	O3—C12—C19—C24	−88.6 (4)
C3—N1—C4—C5	−130.6 (4)	C11—C12—C19—C20	−155.6 (3)
C3—N1—C4—C10	108.6 (4)	C11—C12—C19—C24	29.0 (4)
N1—N2—C11—O2	−10.3 (5)	C13—C12—C19—C20	−37.7 (4)
N1—N2—C11—C12	176.1 (3)	C13—C12—C19—C24	146.9 (3)
S1—C2—C3—O1	−171.9 (3)	C12—C13—C14—C15	176.8 (4)
S1—C2—C3—N1	7.3 (3)	C18—C13—C14—C15	−0.6 (6)
C1—C2—C3—O1	−48.6 (5)	C12—C13—C18—C17	−177.6 (4)
C1—C2—C3—N1	130.6 (3)	C14—C13—C18—C17	−0.2 (6)
S1—C4—C5—C6A	−49.6 (7)	C13—C14—C15—C16	0.6 (8)
S1—C4—C5—C7	67.9 (5)	C14—C15—C16—C17	0.3 (8)
N1—C4—C5—C6A	63.1 (7)	C15—C16—C17—C18	−1.1 (8)
N1—C4—C5—C7	−179.5 (4)	C16—C17—C18—C13	1.1 (7)
C10—C4—C5—C6A	−173.5 (6)	C12—C19—C20—C21	−177.3 (4)
C10—C4—C5—C7	−56.0 (6)	C24—C19—C20—C21	−1.8 (6)
S1—C4—C10—C9	−68.8 (5)	C12—C19—C24—C23	177.4 (4)
N1—C4—C10—C9	176.7 (4)	C20—C19—C24—C23	2.0 (6)
C5—C4—C10—C9	54.2 (6)	C19—C20—C21—C22	0.4 (7)
C4—C5—C7—C8	61.0 (8)	C20—C21—C22—C23	0.8 (8)
C6A—C5—C7—C8	−177.6 (8)	C21—C22—C23—C24	−0.6 (8)
C5—C7—C8—C9	−59.5 (9)	C22—C23—C24—C19	−0.8 (7)
C7—C8—C9—C10	57.2 (9)		

### Hydrogen-bond geometry (Å, °)

**Table:**

*D*—H···*A*	*D*—H	H···*A*	*D*···*A*	*D*—H···*A*
O3—HO3···O1^i^	0.92 (5)	1.83 (5)	2.744 (4)	175 (5)
N2—HN2···O3	0.86 (3)	2.11 (3)	2.535 (4)	110 (3)
C6A—H6A1···N1	0.96	2.55	2.911 (10)	102
C6A—H6A3···S1	0.96	2.54	2.996 (11)	109
C7—H7B···S1	0.97	2.84	3.255 (7)	107
C14—H14···O3	0.93	2.35	2.726 (5)	103

Symmetry codes: (i) −*x*+1, −*y*, −*z*+1.
